# Understanding Primary Ciliary Dyskinesia: Experience From a Mediterranean Diagnostic Reference Centre

**DOI:** 10.3390/jcm9030810

**Published:** 2020-03-16

**Authors:** Miguel Armengot-Carceller, Ana Reula, Manuel Mata-Roig, Jordi Pérez-Panadés, Lara Milian-Medina, Carmen Carda-Batalla

**Affiliations:** 1 Surgery Department, Faculty of Medicine, University of Valencia, 46010 Valencia, Spain; miguel.armengot@uv.es; 2 ENT Service, University and Polytechnic Hospital La Fe, 46026 Valencia, Spain; 3 Grupo de Biomedicina Molecular, Celular y Genómica IIS La Fe, 46026 Valencia, Spain; 4 Pathology Department, Faculty of Medicine, University of Valencia, 46010 Valencia, Spain; Manuel.Mata@uv.es (M.M.-R.); lara.milian@uv.es (L.M.-M.); carmen.carda@uv.es (C.C.-B.); 5 Subdirección General de Epidemiología, Vigilancia de la Salud y Sanidad Ambiental, Conselleria de Sanitat Universal i Salut Pública, Generalitat Valenciana, 46010 Valencia, Spain; perez_jorpan@gva.es

**Keywords:** standard diagnosis, reference centres, clinical presentation, cilia, primary ciliary dyskinesia

## Abstract

Background: Due to the lack of a gold standard diagnostic test, reference centres with experienced personnel and costly procedures are needed for primary ciliary dyskinesia (PCD) diagnostics. Diagnostic flowcharts always start with clinical symptoms. Therefore, the aim of this work is to define differential clinical criteria so that only patients clinically compatible with PCD are referred to reference centres. Materials and methods: 18 variables from 476 Mediterranean patients with clinically suspicious PCD were collected. After analysing cilia function and ultrastructure, 89 individuals were diagnosed with PCD and 387 had a negative diagnosis. Simple logistic regression analysis, considering PCD as a dependent variable and the others as independent variables, was done. In order to define the variables that best explain PCD, a step-wise logistic regression model was defined. Aiming to classify individuals as PCD or PCD-like patients, based on variables included in the study, a classification and regression tree (CART) was designed. Results and conclusions: Simple logistic regression analysis shows statistically significant association between age at the beginning of their symptomatology, periodicity, fertility, situs inversus, recurrent otitis, atelectasis, bronchiectasis, chronic productive cough, rhinorrea, rhinusinusitis and recurrent pneumonias, and PCD. The step-wise logistic regression model selected situs inversus, atelectasis, rhinorrea, chronic productive cough, bronchiectasis, recurrent pneumonias, and otitis as PCD predictive variables (82% sensitivity, 88% specificity, and 0.92 Area Under the Curve (AUC)). A decision tree was designed in order to classify new individuals based on pansinusitis, situs inversus, periodicity, rhinorrea, bronchiectasis, and chronic wet cough.

## 1. Introduction 

Primary ciliary dyskinesia (PCD) is a rare disease with an estimated prevalence of 1/20,000–40,000 births (Code Orphanet: ORPHA244). It is a genetically determined condition, characterized by abnormal or absent mobility of motile cilia and flagella. Consequently, PCD patients present a deficient clearance of secretions and detritus from upper and lower airways. Thus, these patients have infections and chronic inflammation of airways, as well as reduced fertility and situs inversus in 40–50% of cases (Kartagener syndrome) [1]. As PCD presents clinical similarities with other chronic respiratory diseases, its diagnostic is often delayed and, consequently, its evolution and prognosis worsen. Symptoms often start from birth, and a daily wet cough, persistent rhinitis, and serous otitis are the most frequent manifestations in children [1].

At present, there is no “gold standard” diagnostic test. However, according to the European Respiratory Society Task Force guidelines [2], PCD patients are diagnosed based on their clinical manifestations, cilia motility pattern, and frequency, measured by high-speed video microscopy (HSVM) [3], cilia ultrastructure, analysed by transmission electron microscopy (TEM) [4], and genetic testing. Nasal nitric oxide (nNO) levels are used as a screening test [1,5], but other diseases, such as adenoid hypertrophy, could also cause low nNO levels in non-PCD patients [6]. Recently, immunofluorescence staining of specific ciliary structure proteins is becoming a potential test that needs to be validated [7,8], and poor sensitivity means that genotyping cannot be used in isolation [6].

PCD testing is expensive and time-consuming and requires an experienced team of clinicians and scientists. It is, therefore, necessary to define specific and differential clinical criteria regarding other causes of chronic respiratory disease, so that only patients clinically compatible with PCD are referred to diagnostic centres [1,2,9]. In this paper, we define a clinical PCD profile, based on the comparison between data from PCD-diagnosed patients and others clinically suspicious of PCD but with negative diagnostic tests (“PCD like”). With this work, we aim to help to identify patients that require PCD testing.

The definition of a specific clinical profile together with the decision tree based on clinical manifestations will help clinicians to know when do they have to refer a patient to a PCD diagnostic reference centre. Although there already exist other studies that aim to identify candidate patients for PCD diagnostic studies [10,11], our work complements studies by defining specific parameters that allow us to differentiate between a group of patients clinically similar to PCD from those who are PCD-confirmed by diagnostic tests.

## 2. Materials and Methods

Samples of nasal epithelial cells for diagnostics and clinical data from patients were collected for the study after informed consent. The study protocol complied with the ethical guidelines of the 1975 Declaration of Helsinki [12]. Before starting the PCD diagnostic pathway, cystic fibrosis and alpha-1-antitrypsin deficiency were discarded. Diagnostics were established with the study of ciliary motility using HSMV and ciliary structure by TEM, according to the criteria established by the European Respiratory Society guidelines^2^. PCD patients were considered positive when having abnormal ciliary motility by HSVM and presented a TEM defect. Additionally, patients without the obvious TEM defect, who presented abnormal ciliary motility in three different HSVM analyses (when patients were free of infection), with strong clinical history, and low nNO in (those who had the measure), were also considered in the PCD group. In contrast, patients with normal HSVM and TEM tests were considered in PCD-like group.

### 2.1. Study Population and Clinical Data

A total of 18 variables from 476 Mediterranean patients clinically suspicious of PCD were collected in the Valencian PCD Reference Centre from 2005 to 2018: gender, age at the beginning of their clinical symptomatology (younger or older than 2 years old), familiar history of respiratory diseases, periodicity, fertility problems, situs inversus, otitis, immunodeficiency, asthma, atelectasis, bronchiectasis, chronic productive cough, rhinorrhea, rhinosinusitis, pansinusitis, pneumonias, and nasal polyposis. Additionally, tobacco was included in the study as it is a standard variable included in the majority of respiratory disease studies. After studying ciliary motility frequencies and patterns and ciliary ultrastructure, 89 PCD cases were confirmed and 387 PCD-like cases were obtained.

Male infertility was determined, only in adults, by a spermiogram after obtaining patients’ informed consent. By contrast, females were considered infertile after 3 years of failure in their attempts to become pregnant [3,13]. Immunodeficiency was considered by immunoglobulins, blood cell counting, and C3 and C4 complement determination. Functional tests, such as vaccine titers, were not considered for immunodeficiency diagnostics. Asthma was considered, according to GEMA 4.4 Guide (Spanish Guide for asthma management) [14]. Family history of respiratory diseases were considered when they presented chronic bronchopulmonary or rhinosinusal disease with unclear aetiology. Bronchiectasis and atelectasis were characterized according to Kennedy et al., 2007, [15] and rhinosinusitis, according to the European Position Paper on Rhinosinusitis and Nasal Polyps [16]. A partial or total occupation of all sinus, what is known as pansinusitis, was categorized according to the Beguinon et al., 2019, evaluation of PCD patients [17].

### 2.2. Data Analysis

Statistical analysis was carried out with R for windows software (R Foundation for Statistical Computing, Vienna, Austria) [18]. A 5% probability of rejecting a null hypothesis when it is true (α = 0.05) was established in all tests. For all categorical variables, contingency tables were used, which reflected the number of data observed in each category and each group. Simple logistic regression (SLR) analysis, considering PCD as the dependent variable and the others as independent variables, was done with all variables. In some variables, there were missed values because particular clinical data from some patients are unknown. These missed values were not taken into account for statistical analysis.

### 2.3. Multivariate Logistic Regression Model

A multivariate logistic regression analysis was carried out. All variables were entered into the model individually, and a step-wise selection was made to identify and select the significant predictors of PCD. Moreover, the influence of each significant variable on PCD diagnosis was assessed. Finally, a receiver operating characteristic (ROC) curve showing sensitivity, specificity, and overall accuracy was used to interpret significant predictors [19]. Discrimination was considered moderate if Area Under the Curve (AUC) 0.6–0.8 and good if AUC > 0.8 [20]. The Hosmer–Lemeshow goodness-of-fit test was used to assess the calibration of the model, indicating a result of <0.05, which the predicted probabilities, and the current outcome agrees poorly [21].

### 2.4. Classification and Regression Tree (CART)

Aiming to classify individuals as PCD or PCD-like patients based on variables included in the study, a multivariate data analysis was carried out [22]. A classification and regression tree (CART) was designed, which is based on decision rules that appear in a binary tree manner [23,24]. The leaf nodes of the tree contain an output variable, which is used to make a decision. In order to search for the best algorithm, the program starts by growing an overly large tree using forward selection. At each step, it finds the best split and grows until reaching all terminal nodes. It then prunes the tree back, creating a nested sequence of trees, decreasing in complexity [25].

## 3. Results

### 3.1. Study Population

#### 3.1.1. Demographic Characteristics

From the initial population of 476 PCD clinically suspicious cases, 89 individuals were diagnosed with PCD (19%) and 387 (81%) had a negative diagnosis (Table 1). The age range and the median value was 19 (0–76) for the PCD group and 13 (2–83) for the PCD-like group. Additionally, we had 116 adults in the PCD-like group and 45 adults in the PCD group.

In our PCD population, 18.6% had immotile cilia, 34.6% had stiff ciliary movement, and 46.8% had low frequency and uncoordinated movement when analysing using HSVM. Additionally, 20% of our PCD patients had normal ultrastructure, 14.1% undetermined defects (ciliary ultrastructure was unable to be determined because there were insufficient ciliary numbers, inadequate orientation, bad quality of the simple, etc. after sampling repetition), 18.6% combined Inner Dynein Arm (IDA) and Outer Dynein Arm (ODA) defects, 38.6% partial IDA, ODA, and short arms defects, and 9.2% presented other abnormalities (such as axoneme disorganization) when studying ciliary ultrastructure by TEM.

A total of 53% of PCD-like individuals were males, while 47% were females. A total of 52% of PCD patients were males, whereas 48% were females. SLR has a *p*-value of 0.861, indicating that gender is not significantly related to PCD.

#### 3.1.2. Tobacco

A total of 97% PCD-like patients were non-smokers, having the same percentages as the PCD patients group. As expected, SLR had a *p*-value of 0.692, so was not significantly related to PCD.

#### 3.1.3. Age at the Beginning of Symptomatology

Based on the age of the patients at the beginning of their clinical manifestations, two groups were made: younger than 2 years old and older than 2 years old. A tota, of 81% of PCD-like patients were under 2 years, whereas 19% were older than 2 years. A total of 98% of PCD patients were under 2 years and 2% of them were older than 2 years. A *p*-value of <0.001 in SLR shows that, in the great majority of PCD patients, symptoms started before they were 2 years old.

#### 3.1.4. Family History of Respiratory Diseases

A total of 63% PCD-like individuals had no familiar history of respiratory diseases and 53% of PCD ones also had no familiar history of respiratory diseases. SLR had a *p*-value of 0.076, indicating that familiar history is not significantly related to PCD.

#### 3.1.5. Periodicity

While 92% of PCD patients presented their clinical symptoms in a perennial manner, in PCD-like cases, 50% of them presented their symptoms perennially. A *p*-value of <0.001 indicates that the variable periodicity is significantly related with PCD; the risk of having perennial symptoms being 11 times higher in PCD patients than in PCD-like patients.

#### 3.1.6. Fertility Problems

Out of 89 PCD patients, 60 did not proceed with any information about their fertility due to the fact that they were minors or they did not consent to spermiogram realization. Therefore, in our cohort, we had 66% with and 34% without fertility problems. On the other hand, 53% of PCD like-patients presented fertility problems and 47% of them had no problems related to fertility. A *p*-value of 0.029 indicated that fertility is significantly related with PCD, and the risk of having fertility problems in PCD patients was 67% higher than in PCD-like patiens.

#### 3.1.7. Situs Inversus

While 30% of PCD patients presented situs inversus, only 4% of PCD-like patients presented this characteristic. SLR had a *p*-value of <0.001, indicating that the probability of having situs inversus is 11 times higher in PCD patients than in PCD-like patients. 

#### 3.1.8. Chronic Otitis Media

Results show that 33% of PCD-like patients suffered from chronic otits media during their life. In contrast, 68% of PCD patients presented chronic otitis media. SLR had a *p*-value of <0.001, indicating that variable otitis is significantly related to PCD and the probability of having otitis in PCD patients is 4.38 times higher than in PCD-like patients. 

#### 3.1.9. Immunodeficiency

A total of 3% of PCD-like patients had immunodeficiency. However, as none of the PCD patients presented immunodeficiency, SLR analysis did not make sense.

#### 3.1.10. Asthma

A total of 28% of PCD-like patients had asthma. Similarly, 27% of PCD patients presented with asthma. SLR had a *p*-value of 0.785, indicating that asthma is not significantly related to PCD.

#### 3.1.11. Atelectasis

Our results show that 7% of PCD-like patients presented atelectasis at any time. In contrast, 28% of PCD patients had atelectasis. SLR had a *p*-value of < 0.001, indicating that atelectasis is significantly related to PCD, and the risk of having atelectasis in PCD patients was 5.27 times higher than in PCD-like patients. 

#### 3.1.12. Bronchiectasis

A total of 29% of PCD-like patients presented bronchiectasis. In contrast, 68% of PCD patients had bronchiectasis. SLR analysis had a *p*-value of <0.001, indicating that the variable bronchiectasis is significantly related to PCD. The risk of suffering bronchiectasis is 5.37 times higher in PCD-diagnosed patients.

Bronchiectasis are clinical manifestations that are not present from birth and appear with age [1]. Our cohort of PCD-confirmed patients support this fact, as the mean age of PCD patients with bronchiectasis was 31.2, and for PCD patients without this clinical symptom, the mean age was 15.8. The *p*-value of the t-test was <0.001, indicating that the mean age of PCD-patients with bronchiectasis was significantly higher than PCD-patients without bronchiectasis.

#### 3.1.13. Chronic Productive Cough

Results show that 66% of PCD-like patients suffered a chronic productive cough. In contrast, 97% of PCD patients presented a chronic productive cough. SLR with a *p*-value of <0.001 indicates that a chronic productive cough is significantly related to PCD. The risk of presenting a chronic productive cough was 14.56 times higher in PCD patients than in PCD-like patients.

#### 3.1.14. Rhinorrhea

A total of 45% of PCD-like patients presented rhinorrhea. In contrast, 93% of PCD diagnosed patients presented this clinical manifestation. A *p*-value of <0.001 in the SLR indicates that rhinorrhea is significantly related to PCD and is 17 times higher in PCD diagnosed patients.

#### 3.1.15. Rhinosinusitis

A total of 17% of PCD-like patients had chronic rhinosinusitis. On the other hand, 62% of PCD patients had chronic rhinosinusitis. SLR analysis had a *p*-value of <0.001, indicating that rhinosinusitis is significantly related with PCD, and the risk was 7.65 times higher in PCD patients than in PCD-like patients.

#### 3.1.16. Pansinusitis

From 89 PCD patients, 68 patients had no information about pansinusitis (children under 14 do not have a complete sinus formation and, therefore, maxillofacial computed axial tomography does not proceed in these patients). Of 21 patients with pansinusitis information, our cohort had 95% of PCD patients with pansinusitis. In contrast, none of the 89 analyzed PCD-like patients presented pansinusitis. In this case, SLR does not make sense because of the disequilibrium and low number of PCD patients.

#### 3.1.17. Pneumonias

Results show that 39% of PCD-like patients and 73% of PCD patients suffered recurrent pneumonias. SLR with a *p*-value of <0.001 indicates that recurrent pneumonias are significantly related to PCD. The risk of this clinical manifestation was 4.19 times higher in PCD patients than in PCD-like patients. 

#### 3.1.18. Nasal Polyposis

Results show that only 4% of PCD-like patients and 2% of PCD patients presented nasal polyposis. SLR had a *p*-value of 0.497, indicating that nasal polyposis is not significantly related to PCD.

### 3.2. Stepwise Logistic Regression Model

Only individuals that started their symptomatology from infancy were considered for the step-wise logistic regression model, as there were not enough individuals that started in adulthood. From the 18 variables included in the program, 7 were selected for the best logistic regression model (Table 2). These predictors were situs inversus, atelectasis, rhinorrhea, chronic productive cough, bronchiectasis, recurrent cases of pneumonia, and otitis (ordered by the odds ratio value); *p*-values indicate that all variables were statistically significant.

**Figure 1:**
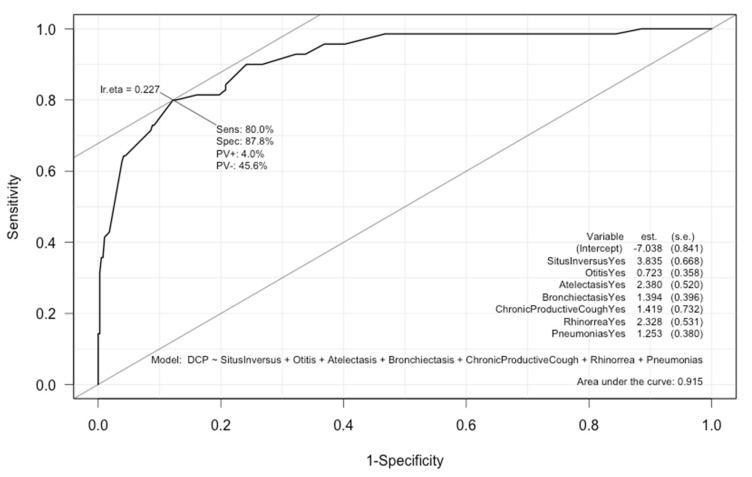
Receiver operating characteristic (ROC) curve for the best prediction model. Sensitivity 80%, specificity 88%, Area Under the Curve (AUC) 0.92.

The Hosmer–Lemeshow test showed a good agreement between the predicted probabilities and the actual outcome (*p* = 0.1182). The sensitivity (proportion of PCD patients correctly identified) and specificity (proportion of PCD-like patients correctly identified) of the model were 80% and 88%, respectively. The discriminant ability (AUC) of this model was 0.92, indicating a good discriminative capacity (Figure 1).

### 3.3. Classification and Regression Tree Model

**Figure 2:**
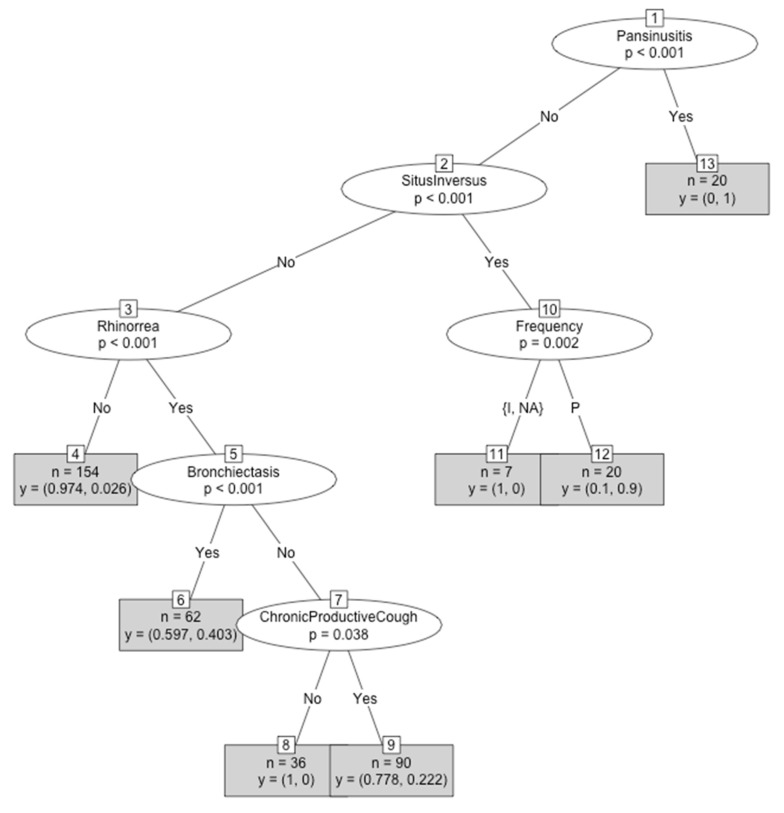
Classification and regression tree (CART) for the classification of individuals as PCD or PCD-like by using clinical variables as classificatory criteria. Intermittent (I), perennial (P), and not-applicable (NA).

From all the variables included in the program, six were selected for the best decision tree (pansinusitis, situs inversus, periodicity, rhinorrhea, bronchiectasis, and chronic wet cough). Only individuals who started their symptomatology from infancy were considered for the CART design, as there were not enough individuals that started in adulthood. Based on these mentioned variables, an individual could be classified as a PCD patient or a PCD-like patient. As previously mentioned, pansinusitis only can be applied in patients older than 14 years old, as computed axial tomography findings could only be considered from this age. In each node (y = p1, p2), the probability of being PCD-like (p1) or PCD (p2) is specified (Figure 2). The number of patients belonging to each final group is also written.

- An individual with pansinusitis will be classified in group 13 (*n* = 20), with a 100% probability of being PCD and 0% probability of being PCD-like.
- An individual without pansinusitis that has situs inversus and intermittent periodicity will be classified in group 11 (*n* = 7) with a 100% probability of being PCD-like.
- An individual without pansinusitis that presents situs inversus and a perennial periodicity will be classified in group 12 (*n* = 20), with a 10% probability of being PCD-like and a 90% probability of being PCD.
- An individual without pansinusitis, situs inversus, and rhinorrhea will be classified in the fourth group (*n* = 154), with a 97.4% probability of being PCD-like and a 2.6% probability of being PCD.
- An individual without pansinusitis and situs inversus who presents rhinorrhea and bronchiectasis will be classified in the sixth group (*n* = 62), with a 59.7% probability of being PCD-like and a 40.3% probability of being PCD.
- An individual without pansinusitis, situs inversus, bronchiectasis, and chronic wet cough who presents rhinorrhea will be classified in group 8 (*n* = 36), with a 100% probability of being PCD-like.
- An individual without pansinusitis, situs inversus, and bronchiectasis who presents rhinorrhea and chronic wet cough will be classified in group 9 (*n* = 90), with a 77.8% probability of being PCD-like and a 22.2% probability of being PCD.

## 4. Discussion

From our experience as a reference centre, we strongly believe that patient clinics are determinant for diagnostics. As mentioned before, expert personnel and expensive techniques are required, so only a compatible clinical history justifies the study [26]. Therefore, it is necessary that primary care clinicians, paediatricians, Ear, Nose and Throat clinicians (ENTs), and pulmonologists know PCD clinical compatible symptoms, in order to select which patients to refer to PCD centres. With this aim, we defined a clinical PCD profile, focused on symptoms that are significantly different in PCD-confirmed cases and PCD-like cases.

The main limitations of the study are that some data were missing for some variables, decreasing statistical test power. Due to the low number of individuals in some groups, we have no comparison between adults and patients groups to demonstrate if there were differences in the logistic regression model and the CART diagram between these age populations. Additionally, all data come from a Spanish reference centre, which can lead to bias.

We demonstrated that the great majority of PCD patients started their symptoms under 2 years old (2 years were selected as a cut-off point because patients had doubts about the neonatal clinic [22]) and they present edtheir symptomatology perennially (it did not differ between seasons). This fact is supported by Leigh et al. [10], who propose the early onset of symptoms and perennial periodicity as characteristics of PCD. Additionally, situs inversus, recurrent otitis, atelectasis, chronic productive cough, rhinorrhea, rhinosinusitis, and recurrent pneumonias are more frequent in PCD patients than in PCD-like patients, indicating that patients with these symptoms are highly suspicious of having PCD.

Immunodeficiency in some moments of its evolution could manifest with symptomatology similar to PCD, and thus we included it in our work to highlight it is an important criterion to discard before referring a patient to PCD diagnostics.

Fertility problems are significantly greater in PCD patients than in PCD-like patients. However, as our cohort was majorly composed of children, these problems were unknown in a great number of patients, which gives us inconclusive results when comparing differences between sexes. Additionally, fertility was the variable with more missing data because many patients did not consent to spermiogram realization for personal reasons. Thus, this variable was not included in the logistic regression model and the CART diagram as it was a variable that introduced bias in our data.

Bronchiectasis is also significantly more frequent in PCD patients than in PCD-like patients, being another PCD-clinical suggesting symptom. However, this clinical manifestation does not appear from birth, and our data demonstrated that this symptom became more frequent in adults than in children with PCD. Thus, bronchiectasis are helpful for PCD-suspicious adults, but it is a manifestation that is less informative in children.

One critical aspect before starting the diagnostic flowchart in a PCD reference centre is to define which are the key clinical manifestations [27,28]. With this aim, the step-wise logistic regression analysis of this work associates situs inversus, atelectasis, rhinorrhea, chronic productive cough, bronchiectasis, recurrent cases of pneumonia, and otitis as PCD predictive variables. These results agree with the four clinical features proposed by Leigh et al. [10] for PCD clinical characterisation, despite differing in the methodology used for diagnostics and statistical analysis.

Behan et al. in the PICADAR (PrImary CiliARy DyskinesiA Rule) study used a similar statistical approach to decide seven variables significantly associated with PCD. They found associations between PCD and situs inversus, neonatal chest symptoms, and hearing problems [11]. Our results are in the same line, but we considered symptoms presenting before 2 years old and chronic otitis media. However, we did not assess admission to the neonatal unit, rhinitis, gestational age, and congenital heart defects in our Mediterranian cohort.

Complementary to our work, Behan et al. [11] in PICADAR and Leigh et al. [10] defined clinical variables that help clinicians suspect PCD. On the one hand, Leigh et al. only included children and adolescents in their study. On the other hand, the PICADAR algorithm design is not sufficient for diagnosing PCD in adult patients as three of their considered items are related to neonates (scoring 6 points out of a maximum of 14). Our diagnostic diagrams arise not only in new-borns or paediatric patients, but also in adult clinics. This is a relevant point, as there are still many adults with PCD without a correct diagnostic and not all of them remember to register or have registered their manifestations when they born, so our method, which was also based in exploratory findings (such as rhinosinusal and pulmonary Computed Tomgraphy (CT)), is a valuable tool for adult patients. Additionally, in this work, we defined a classificatory tree that could help clinicians to know the probability of having a PCD patient, according to their clinical manifestations, based on our experience [23].

## 5. Conclusions

- SLR analysis shows a statistically significant association between some explicative variables and PCD: age at the beginning of their symptomatology, periodicity, fertility, situs inversus, recurrent otitis, atelectasis, bronchiectasis, chronic productive cough, rhinorrhea, rhinosinusitis, and recurrent pneumonias.
- Bronchiectasis is significantly more frequent in adults than in children with PCD.
- A step-wise logistic regression model selected situs inversus, atelectasis, rhinorrhea, chronic productive cough, bronchiectasis, recurrent pneumonias, and otitis as PCD predictive variables (from the most to the least important predicting factor), designing a model with 82% sensitivity, 88% specificity, and 0.92 AUC. Combination of all these clinical symptoms in the same patient determines a high probability of having PCD.
- A decision tree was designed in order to classify new individuals based on different clinical manifestations: pansinusitis, situs inversus, periodicity, rhinorrhea, bronchiectasis, and chronic wet cough.

## Figures and Tables

**Table 1:** Demographical and clinical symptom characteristics of the study populations.

	Total	PCD	PCD-Like	Adjusted OR (95% CI)	*p*-Value
**Subjects (*n*)**	476 (1)	89 (0.19)	387 (0.81)	-	-
**Gender**					
Male	250 (0.53)	46 (0.52)	204 (0.53)	1.04 (0.66–1.65)	0.861
Female	226 (0.47)	43 (0,48)	183 (0,47)		
**Tobacco**					
Smoker	13 (0.03)	3 (0.03)	10 (0.03)	1.31 (0.35–4.87)	0.692
Non-smoker	462 (0.97)	86 (0.97)	376 (0.97)		
**Age at the Beginning of Symptomatology**					
Older than 2 years old	73 (0.16)	2 (0.02)	71 (0.19)	10.23 (2.46–42.54)	**<0.001**
Younger than 2 years old	389 (0.84)	87 (0.98)	302 (0.81)		
**Family History of Respiratory Diseases**					
Yes	185 (0.39)	42 (0.47)	143 (0.37)	1.52 (0.96–2.43)	0.076
No	291 (0.61)	47 (0.53)	244 (0.63)		
**Periodicity**					
Intermittent	186 (0.42)	7 (0.08)	179 (0.50)	11.65 (5.24–25.90)	**<0.001**
Perennial	262 (0.58)	82 (0.92)	180 (0.50)		
**Fertility Problems**					
Yes	61 (0.56)	19 (0.66)	42 (0.53)	1.67 (0.69–4.05)	**0.029**
No	47 (0.44)	10 (0.34)	37 (0.47)		
**Situs Inversus**					
Yes	40 (0.08)	26 (0.30)	14 (0.04)	11.17 (5.53–22.57)	**<0.001**
No	435 (0.92)	62 (0.70)	373 (0.96)		
**Chronic Otitis Media**					
Yes	185 (0.39)	58 (0.68)	127 (0.33)	4.38 (2.65–7.25)	**<0.001**
No	286 (0.61)	27 (0.32)	259 (0.67)		
**Immunodeficiency**					
Yes	12 (0.03)	0 (0)	12 (0.03)	-	-
No	464 (0.97)	89 (1)	375 (0.97)		
**Asthma**					
Yes	130 (0.28)	22 (0.27)	108 (0.28)	0.93 (0.54–1.59)	0.785
No	339 (0.72)	61 (0.73)	278 (0.72)		
**Atelectasis**					
Yes	47 (0.10)	21 (0.28)	26 (0.07)	5.27 (2.78–10.01)	**<0.001**
No	414 (0.90)	55 (0.72)	359 (0.93)		
**Bronchiectasis**					
Yes	165 (0.35)	54 (0.68)	111 (0.29)	5.37 (3.18–9.06)	**<0.001**
No	301 (0.65)	25 (0.32)	276 (0.71)		
**Chronic Productive Cough**					
Yes	342 (0.72)	86 (0.97)	256 (0.66)	14.56 (4.52–46.92)	**<0.001**
No	133 (0.28)	3 (0.03)	130 (0.34)		
**Rhinorrhea**					
Yes	255 (0.54)	83 (0.93)	172 (0.45)	17.21 (7.34–40.37)	**<0.001**
No	220 (0.46)	6 (0.07)	214 (0.55)		
**Rhinosinusitis**					
Yes	120 (0.25)	53 (0.62)	67 (0.17)	7.65 (4.60–12.71)	**<0.001**
No	352 (0.75)	33 (0.38)	319 (0.83)		
**Pansinusitis**					
Yes	20 (0.18)	20 (0.95)	0 (0)	-	-
No	90 (0.82)	1 (0.05)	89 (1)		
**Pneumonias**					
Yes	217 (0.46)	65 (0.73)	152 (0.39)	4.19 (2.51–6.98)	**<0.001**
No	259 (0.54)	24 (0.27)	235 (0.61)		
**Nasal Polyposis**					
Yes	16 (0.03)	2 (0.02)	14 (0.04)	0.61 (0.14–2.74)	0.497
No	460 (0.97)	87 (0.98)	373 (0.96)		

Primary Ciliary Dyskinesia (PCD), bold format means the statistically significant *p*-values.

**Table 2:** Factors that best predict primary ciliary dyskinesia selected by step-wise logistic regression. Regression coefficient, adjusted odds ratio (by the others variables included), and test *p*-value are included.

	Regression Coefficient	Adjusted OR (95% CI)	*p*-Value
**Situs Inversus**	3.835	46.29 (12.51–171.33)	<0.001
**Chronic Otitis Media**	0.723	2.06 (1.02–4.15)	0.043
**Atelectasis**	2.380	10.81 (3.9–29.97)	<0.001
**Bronchiectasis**	1.394	4.03 (1.85–8.76)	<0.001
**Chronic Productive Cough**	1.419	4.13 (0.98–17.34)	0.032
**Rhinorrhea**	2.328	10.26 (3.63–29.03)	<0.001
**Pneumonias**	1.253	3.5 (1.66–7.38)	<0.001
